# A review of experimental and clinical studies on the therapeutic effects of pomegranate (*Punica granatum*) on non‐alcoholic fatty liver disease: Focus on oxidative stress and inflammation

**DOI:** 10.1002/fsn3.3713

**Published:** 2023-09-21

**Authors:** Mohammad Yassin Zamanian, Mehraveh Sadeghi Ivraghi, Lusine G. Khachatryan, Diana E. Vadiyan, Hanie Yavarpour Bali, Maryam Golmohammadi

**Affiliations:** ^1^ Department of Physiology, School of Medicine Hamadan University of Medical Sciences Hamadan Iran; ^2^ Department of Pharmacology and Toxicology, School of Pharmacy Hamadan University of Medical Sciences Hamadan Iran; ^3^ School of Medicine Qazvin University of Medical Sciences Qazvin Iran; ^4^ Department of Pediatric Diseases, N.F. Filatov Clinical Institute of Children's Health I.M. Sechenov First Moscow State Medical University (Sechenov University) Moscow Russia; ^5^ Institute of Dentistry, Department of Pediatric, Preventive Dentistry and Orthodontics I.M. Sechenov First Moscow State Medical University (Sechenov University) Moscow Russia; ^6^ Student Research Committee Babol University of Medical Sciences Babol Iran; ^7^ School of Medicine Shahid Beheshti University of Medical Sciences Tehran Iran

**Keywords:** anti‐inflammatory, antioxidants, herbal medicine, liver enzymes, natural polyphenols

## Abstract

Non‐alcoholic fatty liver disease (NAFLD) is frequently linked to metabolic disorders and is prevalent in obese and diabetic patients. The pathophysiology of NAFLD involves multiple factors, including insulin resistance (IR), oxidative stress (OS), inflammation, and genetic predisposition. Recently, there has been an emphasis on the use of herbal remedies with many people around the world resorting to phytonutrients or nutraceuticals for treatment of numerous health challenges in various national healthcare settings. Pomegranate (*Punica granatum*) parts, such as juice, peel, seed and flower, have high polyphenol content and is well known for its antioxidant capabilities. Pomegranate polyphenols, such as hydrolyzable tannins, anthocyanins, and flavonoids, have high antioxidant capabilities that can help lower the OS and inflammation associated with NAFLD. The study aimed to investigate whether pomegranate parts could attenuate OS, inflammation, and other risk factors associated with NAFLD, and ultimately prevent the development of the disease. The findings of this study revealed that: 1. pomegranate juice contains hypoglycemic qualities that can assist manage blood sugar levels, which is vital for avoiding and treating NAFLD. 2. Polyphenols from pomegranate flowers increase paraoxonase 1 (PON1) mRNA and protein levels in the liver, which can help protect liver enzymes and prevent NAFLD. 3. Punicalagin (PU) is one of the major ellagitannins found in pomegranate, and PU‐enriched pomegranate extract (PE) has been shown to inhibit HFD‐induced hyperlipidemia and hepatic lipid deposition in rats. 4. Pomegranate fruit consumption, which is high in antioxidants, can decrease the activity of AST and ALT (markers of liver damage), lower TNF‐α (a marker of inflammation), and improve overall antioxidant capacity in NAFLD patients. Overall, the polyphenols in pomegranate extracts have antioxidant, anti‐inflammatory, hypoglycemic, and protective effects on liver enzymes, which can help prevent and manage NAFLD effects on liver enzymes, which can help prevent and manage NAFLD.

## Graphical abstract

Non‐alcoholic fatty liver disease (NAFLD) is a common chronic liver disease characterized by fat accumulation in the liver. Pomegranate has been shown to have beneficial effects on insulin resistance and obesity, which are linked to NAFLD pathogenesis. A study investigated the potential protective effect of pomegranate peel extract (PPE) against oxidative stress in the liver of rats with NAFLD. The results showed that PPE improved liver enzymes, inhibited lipogenesis, and enhanced the cellular redox status in the liver tissue of rats with NAFLD.

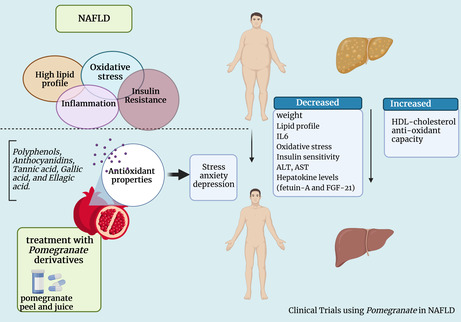

## 1 INTRODUCTION

Non‐alcoholic fatty liver disease (NAFLD) is quickly evolving into a more significant public health concern in the field of liver disease (Muthiah et al., 2022). NAFLD affects about a quarter of the population worldwide, and cases are steadily increasing (Younossi et al., 2019). Men are at a higher risk for NAFLD than women, although the risk for postmenopausal women is similar to that of men (Lee et al., 2019; Pan & Fallon, 2014). The disease is defined by the accumulation of fat in over 5% of liver cells, as determined through histological or radiological methods. Secondary factors like alcohol consumption, viral hepatitis, or inherited liver diseases are not the cause of this condition (Loomba et al., 2021; Pervez et al., 2023). The development of NAFLD involves the excessive buildup of triglycerides (TGs) in liver cells. In 25% of patients, this can progress to non‐alcoholic steatohepatitis (NASH) due to oxidative stress (OS). NASH is an intensified version of NAFLD that is marked by progressive liver cell damage (ballooning and inflammation) and can lead to hepatic fibrosis and concomitant issues, such as liver cancer (Lonardo et al., 2022; Noori et al., 2017). NAFLD is most commonly found in populations with a high incidence of metabolic syndrome. Metabolic risk factors, such as adiposity, type 2 diabetes, cardiovascular disease (CVD), and high cholesterol levels are the primary contributors to the development of the condition (Fotbolcu & Zorlu, 2016; Muzurović et al., 2022; Xu et al., 2016). Eating red meat and drinking soft drinks while not consuming enough fruits and vegetables may increase the likelihood of developing the disease. Moreover, a diet high in carbohydrates and fat can worsen insulin resistance (IR), which is a key factor in developing NAFLD (He et al., 2020; Madan et al., 2006; Rahimi‐Sakak et al., 2022). Furthermore, genetics hold a key position in increasing the likelihood of developing the condition and can result in more severe liver damage (Kozlitina et al., 2014; Seko et al., 2022).

Liver inflammation, increased OS, and fat accumulation are the key factors in developing NAFLD and are linked to IR. OS can increase inflammation and play a significant role in IR (An et al., 2021; Hurrle & Hsu, 2017). The byproducts of oxidation are toxic and can advance the production of pro‐inflammatory cell signaling molecules, such as tumor necrosis factor‐alpha (TNF‐α), interleukin‐6 (IL‐6), and fibrinogen, leading to fibrosis as well as inflammation (Chen et al., 2020; Edmison & McCullough, 2007; Madan et al., 2006). As a result, antioxidant and anti‐inflammatory treatments may benefit liver diseases. Lifestyle changes, including targeted weight loss, are the primary approach to treating NAFLD. However, some obese patients may develop musculoskeletal issues that limit their ability to engage in physical activity (Wong & Singal, 2019). Currently, there are no approved drugs specifically for treating the condition. Treatment options focus on preventative measures, lifestyle changes, and physical activity (Mascaró et al., 2021). As there are still no safe and effective medications available, the search for potential and promising drugs for NAFLD remains a priority. Natural herbal products are a rich resource and have received special attention in the development of drugs for NAFLD (Dang et al., 2019, 2020; Ying et al., 2021).

*Punica granatum* (pomegranate) has attracted the attention of researchers due to its potential for the use in medicine and in the food industry. The beneficial compounds found in pomegranates are not only present in the edible part of the fruit but also in non‐edible parts such as leaves, buds, bark, seeds, peel, and blossoms (Elbakry et al., 2023; Xiang et al., 2022). Pomegranate supplementation has been demonstrated in multiple studies to have neuroprotective (George et al., 2022), anti‐inflammatory (Sayed et al., 2022), hepatoprotective (Al‐Gareeb & Mohammed, 2022; Diab et al., 2022), and antioxidant effects (Lotfi et al., 2022). The therapeutic properties of pomegranates are due to the presence of bioactive compounds, such as anthocyanins, gallic acid, catechin, vitamin C, punicalin, and quercetin (Maphetu et al., 2022). Previous studies have demonstrated the positive impact of pomegranate on IR as well as adiposity, both of which are linked to causing NAFLD. As such, pomegranate may be beneficial in improving NAFLD (Al‐Shaaibi et al., 2016; Goodarzi et al., 2021; Zou et al., 2014). Pomegranate extract's potential to prevent OS has been connected to its capacity to protect the liver from NAFLD‐induced inflammation in overweight rats (Noori et al., 2017). Further research has shown that pomegranate juice can function as a shield in preventing the onset of NAFLD and enhancing lipid profiles. As such, individuals at risk for fatty liver or high cholesterol may benefit from incorporating pomegranate juice into their diets (Hassan et al., 2018). Additionally, clinical research has indicated that consuming antioxidant‐rich pomegranates may be a valuable way to enhance the antioxidant levels of NAFLD patients following a low‐calorie diet (Ekhlasi et al., 2013, 2015).

This review examines the primary ways in which OS and inflammation contribute to the emergence of NAFLD and reports on the impact of pomegranate supplementation on these factors. The goal is to identify the potential targets for new treatments for this condition.

## 2 CHEMICAL COMPOSITION OF *POMEGRANATES*

The pomegranate tree, scientifically known as *Punica granatum L*., belongs to the Punicaceae family. It is believed to have originated in Iran and is native to central Asia. Due to its ability to thrive in various soil and climate conditions, it is now grown in many regions worldwide (da Silva et al., 2013; Pantiora et al., 2023; Verma et al., 2010). Throughout history, different civilizations have incorporated various components of the pomegranate tree, including its leaves, leaf extract, and flowers, into traditional medicine practices. These cultural traditions have recognized the potential therapeutic benefits of these parts of the pomegranate tree (Melgarejo‐Sánchez et al., 2021). The bark of the *P. granatum* tree is known for its tough, twisted appearance and brown color, and it can grow up to 5 m in height. Traditionally, the bark has been used to treat conditions such as diarrhea, inflammation, and HIV‐1 (Mashavhathakha, 2014; Prasad & Kunnaiah, 2014; Sanna et al., 2021). The layers of light pink, oval‐shaped petals on pomegranate flowers are what make them distinctive (Guerrero‐Solano et al., 2020). They have been traditionally used to treat conditions, such as NAFLD and diabetes, have been proven to exhibit antioxidant, anti‐inflammatory, and antiproliferative characteristics (Bekir et al., 2013; Huang et al., 2005; Wei et al., 2013).

The leaves of the *P. granatum* trees are glossy green and oval‐shaped, growing up to 3 cm in length, and are perennial (Guerrero‐Solano et al., 2020). They are used to treat and manage conditions such as high cholesterol, weight loss, cardiovascular disease, and diabetic nephropathy (Das & Barman, 2012; Rohini et al., 2013; Wang et al., 2018). Studies reported that pomegranate leaf extracts can inhibit adiposity and hyperlipidemia in lab rodents fed a high‐fat diet (HFD), partly by suppressing pancreatic lipase activity and reducing energy intake (Lei et al., 2007). Of all the parts of the *P. granatum* plant, the seeds have been the most extensively studied and documented. They can number in the hundreds and are present inside the fruit, which has red arils surrounding it (Guerrero‐Solano et al., 2020). The edible portion of the pomegranate is the fruit, which contains many seeds ranging from 40 to 100 g/kg. These seeds are often discarded as waste after processing (Baradaran Rahimi et al., 2020). Pomegranate seeds are used for various purposes, including preventing miscarriage (Moga et al., 2021) and promoting bone healing (Mohamed et al., 2022). They have also been found to possess pharmaceutical properties such as antimicrobial activity (Fathi et al., 2020), anti‐cancer (Lepionka et al., 2019), and antioxidant effects (Rojo‐Gutiérrez et al., 2021). The hard pericarp shield of pomegranate fruit peels is identifiable by its orange and greenish color when the fruit is ripe. Inside the peel, arils are separated by a thin membrane. There is a thin membrane dividing the arils inside the peel Pomegranate peels comprise 43% of the entire fruit (Ko et al., 2021). Previously, the peel was considered waste. However, recent research has shown that it contains a wealth of bioactive phytochemicals. These compounds can potentially act as antioxidants, physiological agents, antimicrobial agents, and immune system stimulants (Elbakry et al., 2023; Smaoui et al., 2019). Extracts from the peel have been traditionally used to treat diseases such as type 2 diabetes and carcinoma (Teniente et al., 2023; Zare et al., 2023). The peel of pomegranate has been found to have anti‐inflammatory, hypoglycemic, anti‐apoptotic, and prebiotic properties (Ismail et al., 2014). Pomegranate juice is a popular product that can be extracted from the arils or the whole fruit. The edible portion of the pomegranate makes up 52% of its overall weight, with 78% being juice, and 22% being seeds (Kumar et al., 2022). Pomegranate juice is rich in ascorbic acid and is extracted from the sweet red arils, pulp, and peel of the fruit. It is also a source of phenolic compounds (Ge et al., 2021).

Pomegranate polyphenols are a group of compounds found in pomegranate fruit that have been shown to possess various health benefits, including NAFLD (Goodarzi et al., 2021). These polyphenols include ellagitannins, ellagic acid, and other flavonoids such as quercetin, kaempferol, and luteolin glycosides (Adams et al., 2006; Seeram et al., 2005). The peel of the pomegranate fruit contains particularly high levels of polyphenols, especially ellagitannins (Akhtar et al., 2015). Polyphenols in pomegranate seeds include ellagitannins, which are the most abundant polyphenols in pomegranate (Lipińska et al., 2014). The main ellagitannins found in pomegranate seeds are punicalagins, punicalin A, and punicalin B (Zhang et al., 2009). These ellagitannins are responsible for the antioxidant activity of pomegranate seeds and have been associated with various health benefits (Zhang et al., 2009). Other polyphenols present in pomegranate seeds include flavonoids, such as catechins, epicatechins, and quercetin, as well as anthocyanins, which are responsible for the red color of the seeds (Ranjha et al., 2021; Singh et al., 2018). These polyphenols contribute to the overall antioxidant and health‐promoting properties of pomegranate seeds (Jing et al., 2012). The content analysis reveals that pomegranate juice contains a high concentration of polyphenols, including total polyphenols and specific compounds such as punicalagin and ellagic acid (Seeram et al., 2005).

## 3 PATHOGENESIS OF NAFLD

NAFLD is the most ubiquitous liver disorder. It is defined by fat buildup in the liver without alcohol consumption, steatogenic medication use, or other liver conditions (Sun et al., 2020). NAFLD is frequently viewed as a component of metabolic syndrome due to its close relationship with obesity, non‐insulin‐dependent diabetes mellitus (NIDDM), hypertension, and dyslipidemia (Chalasani et al., 2018). Based on histological characteristics, it can be indexed as either NAFLD or NASH (Leoni et al., 2018). NASH is distinguished by indications of liver cell injury (ballooning and inflammation), regardless of the presence or absence of scarring, as observed in a liver biopsy. Regardless of the presence or absence of scarring, a liver biopsy will reveal signs of liver cell injury (ballooning and inflammation) that distinguish NASH. Twenty percent of NASH patients progress to cirrhosis and have an elevated susceptibility to liver cancer (Sheka et al., 2020). NAFLD is more prevalent in technologically advanced nations, with a worldwide occurrence of nearly 25%. Its occurrence varies by region, being higher in the Middle East and South America, and lower in Africa (Younossi et al., 2019). The prevalence of NASH is indeterminate and is estimated to be between 1.5 and 6.45%, as it is dependent on the accessibility of liver biopsies at referral centers (Younossi et al., 2016).

## 4 OXIDATIVE STRESS AND NAFLD

An OS condition occurs when there is an imbalance between free radicals such as reactive oxygen species (ROS) and antioxidants in the body. This can result in cellular damage and often leads to cell death (Zamanian, Hajizadeh, et al., 2017; Zamanian, Shamsizadeh, et al., 2017).

ROS are highly reactive molecules that can be generated as byproducts of normal cellular metabolism or as a result of exposure to external factors such as environmental toxins, radiation, or certain drugs (Sharma & Kim, 2023; Zamanian, Shamsizadeh, et al., 2017). The main sources of ROS production within cells include mitochondria, NADPH oxidases, and the endoplasmic reticulum (ER) (Bhardwaj et al., 2017; Santos et al., 2009; Zeeshan et al., 2016).

ROS can also react with proteins, causing oxidative modifications that can impair their structure and function (Chakravarti & Chakravarti, 2007). This can lead to protein misfolding, aggregation, and loss of enzymatic activity (Nakamura et al., 2012). Additionally, ROS can also directly induce DNA damage through causing DNA strand breaks, altering DNA bases, and promoting DNA cross‐linking (Srinivas et al., 2019). These DNA lesions can interfere with DNA replication and transcription, leading to genomic instability and potentially contributing to the development of various diseases, including cancer and NAFLD (Paradies et al., 2014; Renaudin, 2021). OS is a critical factor in the pathogenesis and progression of NAFLD (Rives et al., 2020). Excessive accumulation of fat in the liver leads to increased OS, which in turn promotes hepatocyte injury, inflammation, and fibrosis (Bessone et al., 2019; Mohamed et al., 2016). OS can also contribute to IR, which is a key feature of NAFLD (Köroğlu et al., 2016).

Under normal circumstances, cells maintain a basic level of ROS to support redox signaling for diverse functions such as metabolism, cell differentiation and survival, immune defense, and the regulation of transcription factors and epigenetic status (Harris & DeNicola, 2020; Sies & Jones, 2020; Zhou et al., 2013). In the presence of OS, the antioxidant enzyme SOD converts the superoxide radical (O_2_^.−^) into hydrogen peroxide (H_2_O_2_), which is then broken down into oxygen (O_2_) and water (H_2_O) by the enzymes Gpx or CAT (Buettner, 2011; Zamanian, Hajizadeh, et al., 2017). ROS are primarily produced in the mitochondria, peroxisomes, and endoplasmic reticulum (ER) of cells but can also be produced in the cytoplasm. Elevated levels of ROS can damage these organelles, further exacerbating OS and creating a vicious cycle (Das & Roychoudhury, 2014; Yoboue et al., 2018). When the body experiences OS, it activates both enzymatic and non‐enzymatic mechanisms to counteract the production of ROS (Ahmad et al., 2009). Research has shown that these antioxidant mechanisms change during the development of NAFLD. In people with NAFLD, the liver cells' ability to neutralize ROS decreases (Reccia et al., 2017; Świderska et al., 2019). There is a reduction in both enzymatic (CAT, SOD, and GPX) and non‐enzymatic (GSH, TRX, α‐tocopherol, and ubiquinone) antioxidant systems in various bodily fluids and organs (Hajighasem et al., 2019; Irie et al., 2016; Leghi et al., 2015). The high levels of ROS in NAFLD can directly deplete antioxidant molecules and reduce the effectiveness of antioxidant enzymes (Chen et al., 2020).

Paraoxonase‐1 (PON1) is an antioxidant enzyme that is synthesized in the liver. Its primary function is to metabolize and neutralize peroxides and lactones that are associated with lipoproteins (Camps et al., 2021).

PON1 is responsible for hydrolyzing lipid peroxides, which are toxic byproducts of lipid oxidation. By breaking down lipid peroxides, PON1 helps to prevent oxidative damage to cells and tissues (Zargari & Tabaghchi, 2017).

PON1 can prevent the oxidation of LDL, which is a significant risk factor for atherosclerosis and cardiovascular disease. By preventing LDL oxidation, PON1 helps to protect against the development of these diseases (Macharia et al., 2012; Nguyen & Sok, 2003).

The relationship between OS and PON1 is bidirectional (Draganov et al., 2010). On one hand, OS can affect the activity and expression of PON1. OS can lead to the oxidation and inactivation of PON1, reducing its ability to protect against lipid peroxidation and oxidative damage (Nguyen & Sok, 2003). On the other hand, PON1 can also modulate OS by reducing the levels of ROS and lipid peroxides (Nguyen & Sok, 2003). PON1 can hydrolyze lipid peroxides and detoxify oxidized lipids, thereby reducing OS and its detrimental effects (Rosenblat & Aviram, 2009; Wang et al., 2017).

Multiple research studies have demonstrated that the activity of the enzyme PON1 is reduced in various disease states associated with high levels of oxidative stress. These include cardiovascular diseases like atherosclerosis, diabetes mellitus, and chronic liver diseases, such as NAFLD (Milaciu et al., 2019; Selek et al., 2012; Shih & Lusis, 2009; Singh et al., 2007). In NAFLD, impaired lipid metabolism and mitochondrial dysfunction contribute to OS, and PON1 activity is reduced in the liver (Camps & Joven, 2015; Liu et al., 2015). This reduction in PON1 activity may contribute to the progression of NAFLD by allowing increased oxidative damage and inflammation.

A study of 81 individuals with NAFLD found that the concentration of Paraoxonase‐1 in the serum was reduced, indicating higher levels of OS in these patients (Milaciu et al., 2019).

In summary, PON1 is an important enzyme involved in protecting against OS. It can prevent the oxidation of LDL particles and reduce oxidative damage by detoxifying oxidized lipids. However, OS can negatively affect PON1 activity, and reduced PON1 activity may contribute to the development and progression of diseases characterized by high OS, such as NAFLD.

Nuclear factor E2‐related factor 2 (Nrf2) is a vital endogenous antioxidant transcription factor that has been shown to play a critical role in protecting cells from the damaging effects of OS (Zamanian, Giménez‐Llort, et al., 2023).

Under normal conditions, Nrf2 is anchored in the cytoplasm by Kelch‐like ECH‐associated protein 1 (Keap1) and undergoes degradation (Zamanian, Giménez‐Llort, et al., 2023; Zamanian, Parra, et al., 2023). However, when exposed to OS, Nrf2 dissociates from Keap1 and translocates into the nucleus, where it binds to the antioxidant response element (ARE) and initiates the transcription of genes involved in antioxidative defense (Lv et al., 2023; Zamanian, Soltani, et al., 2023). Activation of Nrf2 leads to the upregulation of genes involved in antioxidant defense, detoxification, and repair mechanisms, thereby reducing OS and promoting cell survival (Zamanian, Parra, et al., 2023).

The transcription factor Nrf2 plays a crucial role in regulating the expression of various genes. These genes encode antioxidant enzymes and proteins involved in glutathione synthesis and metabolism. Specifically, Nrf2 controls the expression of the catalytic and modifier subunits of glutamate‐cysteine ligase, an enzyme that catalyzes the first step in glutathione production. Nrf2 also regulates the expression of glutathione peroxidase 2 (GPX2), an enzyme that reduces hydrogen peroxide and organic hydroperoxides by converting glutathione from its reduced to oxidized form (Sharma et al., 2018). In preclinical models, Nrf2 expression is increased in the early stages of NAFLD (Meakin et al., 2014).

NAFLD is associated with increased OS, and Nrf2 activation helps to alleviate this condition (Bataille & Manautou, 2012; Deng et al., 2019). Nrf2 also plays a role in regulating lipid metabolism in NAFLD. It can suppress de novo lipogenesis (DNL) by inhibiting the expression of key lipogenic genes, such as fatty acid synthase (FASN) and stearoyl‐CoA desaturase 1 (SCD1) (Feng et al., 2017; Li et al., 2020). Nrf2 has anti‐inflammatory effects in NAFLD (Li et al., 2021). Nrf2 has the ability to inhibit the production of specific pro‐inflammatory cytokines such as TNF‐α and MCP‐1. By inhibiting the production of these inflammatory signaling molecules, Nrf2 can reduce inflammation in the liver (Meng et al., 2021). Inflammation is a key feature of NAFLD progression, and Nrf2 activation helps to attenuate this inflammatory response (Li et al., 2021). Nrf2 has been shown to have a protective effect against liver fibrosis in NAFLD (Bataille & Manautou, 2012). It can inhibit the activation of hepatic stellate cells, which are responsible for excessive collagen deposition and fibrosis development in the liver (Hu et al., 2019). Overall, Nrf2 plays a critical role in protecting against OS, regulating lipid metabolism, reducing inflammation, and preventing fibrosis in NAFLD. Activation of Nrf2 signaling pathway has emerged as a potential therapeutic strategy for the treatment of NAFLD.

## 5 INFLAMMATION AND NAFLD

Inflammation plays a crucial role in the development and progression of NAFLD (Tilg & Moschen, 2010). It is considered a key driver in the transition from simple steatosis to the more severe form of the disease, NASH, and is associated with the development of liver fibrosis, cirrhosis, and hepatocellular carcinoma (Cannito et al., 2023; Starley et al., 2010).

One of the main triggers of inflammation in NAFLD is OS. OS leads to lipid peroxidation and the release of pro‐inflammatory mediators, such as cytokines (IL‐1β, IL‐6, IL‐12, and TNF‐α) and chemokines, which promote the recruitment and activation of immune cells in the liver (Bruzzì et al., 2018; Darmadi & Ruslie, 2021; Tilg & Moschen, 2010).

Dysfunctional adipose tissue (AT) also contributes to inflammation in NAFLD. Adipose tissue secretes various adipokines, including adiponectin, leptin, resistin, visfatin, and adipsin, which have both pro‐inflammatory and anti‐inflammatory effects. Imbalances in the production of these adipokines, such as decreased adiponectin and increased leptin and resistin levels, contribute to the development of inflammation in NAFLD (Henao‐Mejia et al., 2012; Miele et al., 2021; Tilg & Moschen, 2010; Younossi et al., 2016). Furthermore, infiltration of immune cells, such as macrophages and T cells, into the liver promotes inflammation and the release of pro‐inflammatory cytokines (Harford et al., 2011). These immune cells interact with hepatocytes and promote hepatocyte injury and the production of fibrotic factors, leading to the progression of liver fibrosis (Hammerich et al., 2014).

Immune cells significantly impact the advancement of NAFLD. T cells, a specific type of immune cell, contribute to liver inflammation and the onset of NASH, fibrosis, cirrhosis, and hepatocellular carcinoma (HCC) (Mao et al., 2022). Various subsets of both conventional and unconventional T cells play a role in the development of NAFLD. Inflammation‐related factors and potential therapeutic approaches targeting immune cells in NASH are currently being researched (Mao et al., 2022). B cells, another type of immune cell, are also involved in the development of NAFLD. They can contribute to NAFLD through the production of proinflammatory cytokines and by regulating intrahepatic T cells and macrophages (Barrow et al., 2021). They may be triggered during NAFLD due to an increase in the hepatic production of B‐cell‐activating factor, heightened OS, and the relocation of microbial products originating from the gut (Barrow et al., 2021). Regulatory and IgA^+^ B cells have been emphasized in developing HCC associated with NASH (Barrow et al., 2021). Significant alterations have been observed in the peripheral immune cells of individuals with NAFLD, with some changes being associated with the advancement of NASH and fibrosis resulting from NAFLD. The analysis of peripheral blood immune cells is regarded as a promising non‐invasive technique for monitoring the progression of NASH (Lin & Fan, 2022). In summary, immune cells, particularly T cells and B cells, play a crucial role in the progression of NAFLD. They contribute to liver inflammation, the development of NASH, and related liver complications. Understanding the role of immune cells in NAFLD pathogenesis can help in the development of novel therapeutic approaches targeting these cells.

Additionally, the activation of liver cells that are present in the organ, the attraction of circulating inflammatory cells, and the increased expression of various soluble inflammatory mediators are all hallmarks of NAFLD progression (Haukeland et al., 2006; Paredes‐Turrubiarte et al., 2016). Pro‐inflammatory cytokines such as IL‐12, IL‐6, IL‐1β, and TNF‐α may have a crucial role in the development and progression of NAFLD by promoting IR, OS, liver inflammation, cell death, apoptosis, and liver fibrosis (de Souza Teixeira et al., 2018; Rabelo et al., 2010). Elevated levels of TNF‐α are considered a signature characteristic and key driver of the inflammation associated with obesity and NAFLD. TNF‐α overproduction is believed to be a major causative factor contributing to the development of IR in these conditions (Asrih & Jornayvaz, 2013). TNF‐α is overexpressed in the fat tissue (adipose) and muscle of both obese humans and rodent obesity models. In these tissues, TNF‐α can activate signaling pathways that either promote or inhibit programmed cell death (apoptosis). This leads to activation of the NF‐κB pathway and controls processes like cell survival, inflammation, metabolism, and cytokine secretion. Therefore, excessive production of TNF‐α in adipose tissue and skeletal muscle, two key metabolic tissues, is a consistent feature of obesity in both humans and rodents. Through its downstream effects on NF‐κB signaling, the overabundance of TNF‐α in these tissues contributes to the disturbances in cell viability, inflammation, and metabolism that underlie obesity (Tiniakos et al., 2010). Obese mice lacking the TNF‐α receptor have been found to have better insulin sensitivity compared to their wild‐type counterparts (Wellen & Hotamisligil, 2005). TNF‐α promotes and is activated by IR through NF‐κB and is involved in liver inflammation and metabolic changes (Tiniakos et al., 2010). In addition to its direct proinflammatory effects, TNF‐α can also negate the actions of adiponectin, an anti‐inflammatory cytokine secreted by fat cells (adipocytes). Adiponectin normally improves cellular sensitivity to insulin and promotes fatty acid oxidation. It also has anti‐inflammatory and anti‐lipogenic (fat production inhibiting) effects in the liver (Tiniakos et al., 2010). On the other hand, elevated levels of TNF‐α in the blood are associated with NAFLD disease activity as determined by histological parameters in patients with NAFLD. Similarly, the expression of TNF‐α and TNF‐α receptor genes was increased in liver and fat tissues in patients with NASH (Meli et al., 2014). Conversely, the anti‐inflammatory cytokine IL‐10 plays a crucial role in regulating these processes and promoting liver regeneration following injury (den Boer et al., 2006; El‐Emshaty et al., 2015). When the liver is damaged, OS activates redox‐sensitive transcription factors, including NF‐κB and AP‐1. This activation leads to an inflammatory response and the initiation of cell death pathways in liver cells. In the case of NAFLD, ROS regulate the activation of the NF‐κB pathway by upregulating the expression of the proinflammatory cytokine TNF‐α (Oliveira‐Marques et al., 2009).

NF‐κB, a key regulator of inflammation, plays an important role in controlling the transcription of genes involved in initiating immune and inflammatory responses (de Gregorio et al., 2020). Reducing NF‐κB activity through the use of antioxidants has been suggested as a potential treatment for NASH due to its anti‐inflammatory effects (Flores‐Costa et al., 2020; Komeili‐Movahhed et al., 2022). Moreover, the potential strategy to slow down the progression of NAFLD involves targeting the interaction between NF‐κB and Nrf2. Research has demonstrated that the p65 subunit of NF‐κB inhibits the Nrf2/ARE system by competing with CBP for binding to the transcriptional level (Liu et al., 2008). The transcription factor Nrf2 can inhibit the NF‐κB signaling pathway through several mechanisms. These include preventing NF‐κB from moving into the nucleus and blocking the degradation of IκB‐α, which keeps NF‐κB sequestered in the cytoplasm (Saha et al., 2020).

Understanding the role of inflammation in NAFLD is crucial for the development of targeted therapies. By targeting the inflammatory pathways and immune cells involved in NAFLD, it may be possible to halt or reverse the progression of the disease. Therapeutic interventions that aim to reduce inflammation and restore metabolic homeostasis show promise in the treatment of NAFLD.

**FIGURE 1:**
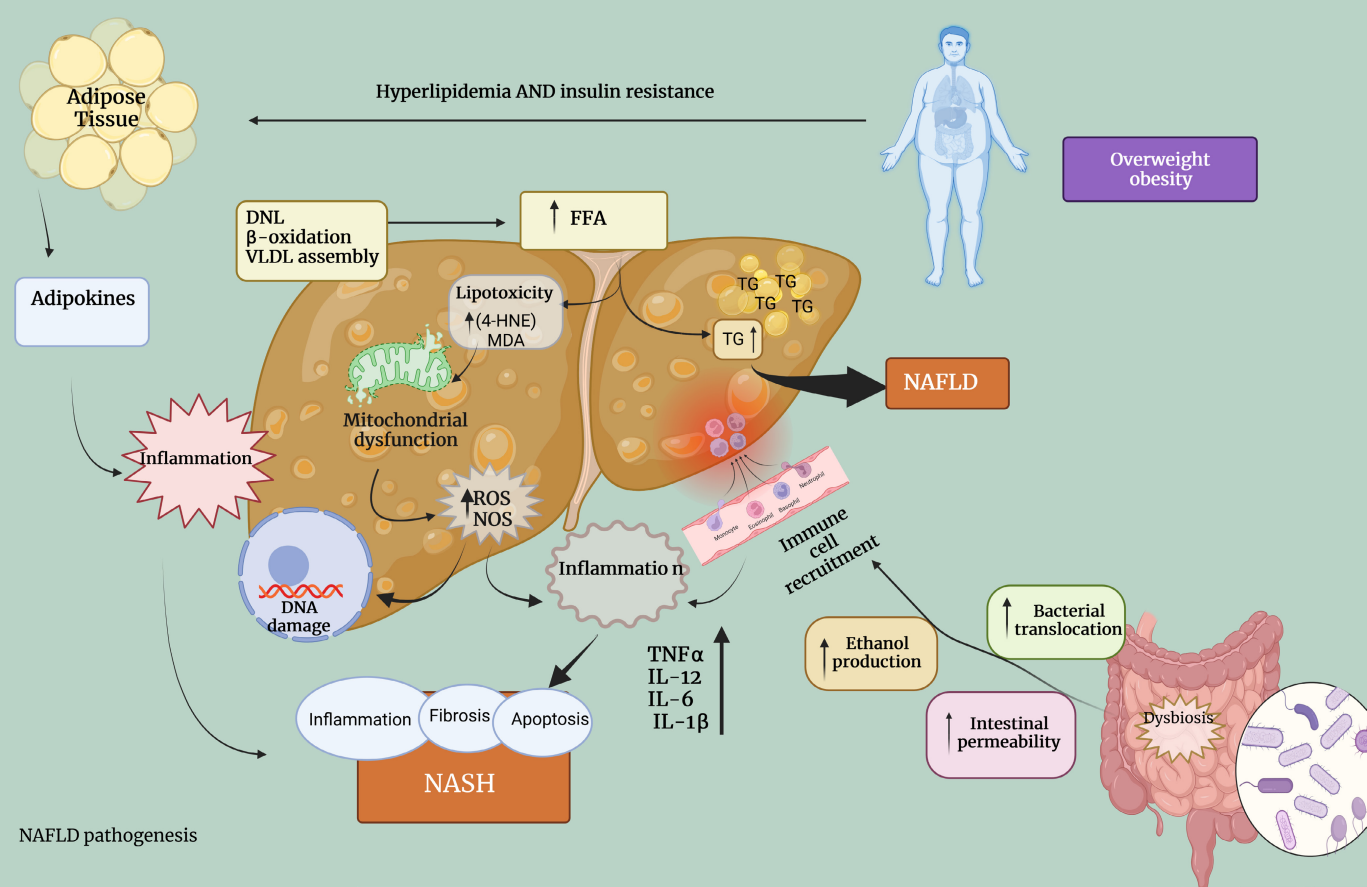
Pathophysiology of NAFLD (Fotbolcu & Zorlu, 2016; Madan et al., 2006; Muzurović et al., 2022; Xu et al., 2016). Diet and environmental factors, combined with obesity, lead to increased levels of fatty acids (FFAs) and cholesterol in the blood. This results in the development of insulin resistance (IR), dysfunctional fat cell proliferation and activity, and changes in the gut microbiome. IR acts on fat tissue worsening dysfunction of fat cells, causing them to break down fat into FFAs and release adipokines and proinflammatory cytokines like IL‐1β, TNF‐α, IL‐6, and IL‐12, which also maintain IR. In the liver, IR increases de novo lipogenesis (DNL). The increased flux of FFAs from fat tissue breakdown and the gut microbiome lead to two outcomes: synthesis and accumulation of triglycerides (TGs), and “toxic” levels of FFAs, free cholesterol, and other lipids. These cause mitochondrial dysfunction with OS and ROS, NOS and ER stress. This results in hepatic inflammation. Also, intestinal permeability can increase, leading to higher levels of molecules like LPS that activate inflammasomes and ER stress, and release pro‐inflammatory cytokines. Genetic factors or epigenetic changes affect fat content, enzyme processes, and inflammation in the liver. This influences whether NAFLD progresses to inflammation and fibrosis (NASH) or remains stable.

Briefly, the pathophysiology of NAFLD is described in Figure 1.

## 6 EXPERIMENTAL STUDIES ON THE THERAPEUTIC EFFECTS OF POMEGRANATE (*PUNICA GRANATUM*) ON NAFLD

A diet rich in fat (HFD) can lead to NAFLD development by encouraging fat buildup within the liver (Pan & An, 2018). Consuming HFD has been found to increase body weight and result in NAFLD, a fatty liver, high blood lipid levels, OS, and raised liver enzyme levels (Huang et al., 2017). Elevated levels of ALT and AST in the blood are indicative of liver cell damage. Studies have shown that individuals with NAFLD tend to have higher levels of these enzymes (Dai et al., 2015; Khosravi et al., 2011).

Al‐Shaaibi et al. found that giving rats pomegranate peel extract (PPE) improved the appearance of their livers, decreased their body weight, improved their liver enzyme levels (AST and ALT), and inhibited the formation of fat. Additionally, PPE improves the cellular redox status by increasing the levels of glutathione (GSH) in the liver tissue. GSH is an important intracellular antioxidant that plays a key role in maintaining cellular homeostasis and protecting against oxidative damage. PPE supplementation restores GSH levels in the liver, which helps to maintain the balance between ROS and antioxidants. In this study, a water extract of PPE was made ready once every 7 days by combining a dry powder with distilled water (15 g dry solids/100 mL) and stored at 4°C until used. Each rat was given PPE orally twice in 7 days at a dose of 2.5 mL/kg body weight. These findings suggest that PPE may have a protective effect against liver damage, which is commonly associated with NAFLD (Al‐Shaaibi et al., 2016).

In another study, Poonam and Rao conducted a study to examine the protective and therapeutic effects of pomegranate seed oil extract (SOE) on NAFLD caused by HFD in rat models. They found that switching from a high‐fat to a low‐fat diet reduced liver damage. However, giving SOE (100 & 200 mg/kg) resulted in a noteworthy decrease in all liver biomarkers examined, indicating that SOE can reverse liver cell damage caused by HFD + fructose. SOE treatment had a protective effect, which was more pronounced in animals given 200 mg/kg of SOE. Giving SOE increased antioxidant levels and decreased lipid peroxidation in treated animals (Singh & Rao, 2022).

According to the study of Ayad et al., supplementation with pomegranate peel extract (PPE) has shown a beneficial effect on lipid profiles and liver function in the context of a HFD. In this study, animals fed a HFD experienced a significant increase in serum levels of cholesterol, TG, ALT, and AST compared to the control group. However, when PPE was administered along with the HFD, there was a remarkable decline in the serum lipid profiles, as well as an improvement in liver function as indicated by the reduction in ALT and AST levels. These findings suggest that PPE supplementation can attenuate the negative effects of a HFD on lipid metabolism and liver health. Furthermore, histological examination of liver tissue showed that animals in the HFD group displayed severe macrovesicular hepatic steatosis, characterized by the accumulation of abnormal amounts of fat in hepatocytes. However, administration of PPE resulted in a clear recovery of the hepatic architecture, with a reduction in the severity of macrovesicular steatosis. This indicates that PPE supplementation can mitigate the development of fatty liver induced by a HFD. The aqueous extract of pomegranate peel was prepared daily by adding 20 g of dry powder to 1000 mL of boiled distilled water (Ayad et al., 2020).

Fabp1, Slc27a1, Cd36, and Fabp4 are all involved in the metabolism of fatty acids. Fabp4 is primarily expressed in fat cells and immune cells called macrophages and plays a significant role in the development of IR and the hardening of arteries in connection with low‐grade and chronic inflammation caused by metabolic factors, known as “metaflammation” (Furuhashi, 2019). Cd36 and Fabp4 work together to control the entry, movement, and processing of fatty acids within cells (Gyamfi et al., 2021). The FATP (fatty acid transport protein) family of proteins, which includes Slc27a1 also known as FATP1, allows cells to absorb long‐chain fatty acids (Li et al., 2022). Pfohl et al. found that supplementing with pomegranate fruit extract (PFE) had a protective effect on the liver against obesity caused by HFD, as shown by reduced liver and body weight in a mouse model. Additionally, PFE supplementation reduced the progression of NAFLD caused by HFD by decreasing the levels of total lipids, TAGs, and FFAs in the liver. Supplementing with PFE while on an HFD significantly reduced the expression of genes involved in fatty acid uptake, including Fabp1, Slc27a1, Cd36, and Fabp4, potentially limiting the influx and accumulation of fatty acids in the liver. Furthermore, PFE supplementation reduced the expression of proinflammatory genes in the liver, including TNF‐α, IL‐6, CSF2Rα, and CCL2 (Pfohl et al., 2021).

Scott and Jamal reported that liver mitochondrial dysfunction occurs before the NAFLD development in a rat model (Rector et al., 2010). Vial and colleagues found that feeding rats HFD resulted in reduced mitochondrial quinine levels and significant changes in mitochondrial lipid composition. This led to a decrease in fatty acid oxidation and an increase in the production of mitochondrial ROS (Vial et al., 2011). Researchers demonstrated that pomegranate extracts can help regulate lipid metabolism in diseases, such as atherosclerosis, NAFLD, and type 2 diabetes, which are closely linked to mitochondrial function (Hou et al., 2019). Furthermore, research has shown that pomegranate peel extract can increase mitochondrial complex IV activity and prevent changes to mitochondrial cristae in the brown adipose tissue of mice on HFD (Echeverria et al., 2021). Overall, pomegranate and its derivatives have potential health benefits and may improve mitochondrial function in various metabolic disorders, such as NAFLD.

Zou and colleagues discovered that treatment with pomegranate husk extract (PHE), particularly at a higher dosage of 150 mg/kg/day, significantly reduced obesity development and improved serum parameters such as cholesterol, adiponectin, and leptin. Punicalagin, which is a major ellagitannin found in pomegranate extract, has been shown to have protective effects against HFD‐induced NAFLD. The study found that punicalagin‐enriched PE administration effectively inhibited HFD‐induced hyperlipidemia and hepatic lipid deposition. The protective effects of punicalagin are attributed to its ability to modulate several key factors involved in the development of NAFLD. First, punicalagin supplementation normalized the expression of pro‐inflammatory cytokines, such as TNF‐α and interleukins (IL‐6 and IL‐4), which are major contributors to NAFLD. Second, punicalagin reduced OS in hepatocytes by activating Nrf2. This led to a decrease in protein oxidation and lipid peroxidation, and an increase in SOD activity, thereby reducing oxidative damage in the liver.

Third, punicalagin improved mitochondrial function, which is closely associated with the progression of NAFLD. In the study, it was found that the treatment resulted in the recovery of ATP levels and inhibited the oxidation of mitochondrial proteins. Additionally, the treatment led to an increase in the activities of mitochondrial complexes II and IV. Punicalagin also decreased the expression of uncoupling protein 2 (UCP2), which is involved in ATP depletion, further improving mitochondrial function. Overall, punicalagin in PE protects against HFD‐induced NAFLD by reducing inflammation, OS, and improving mitochondrial function and lipid metabolism. These findings suggest that punicalagin may be a useful therapeutic nutrient for the treatment of NAFLD (Zou et al., 2014).

PON1 is an enzyme generated by the liver that plays a crucial role in neutralizing harmful oxidants (Aviram & Rosenblat, 2005). A meta‐analysis revealed that PON1 activity could serve as a valuable indicator of NAFLD (Kotani et al., 2021). Another study discovered that serum levels of PON1 were reduced in individuals with NAFLD and that the occurrence of NAFLD was associated with decreased PON1 levels (Milaciu et al., 2019). One study investigated the protective effects and underlying mechanisms of pomegranate flower polyphenols (PFP) on liver function in rats with diabetes and NAFLD induced by high‐calorie feeding and low‐dose intraperitoneal injection of streptozotocin (STZ). (Wei et al., 2013). Peroxisome proliferator‐activated receptors (PPARs) are a sizable family of nuclear transcription factors that need particular ligands to activate them. These receptors play a key role in regulating the expression of genes that control lipid metabolism and storage, influence lipoprotein metabolism, promote adipocyte differentiation, and modulate insulin action (Bogacka et al., 2004; de la Rosa Rodriguez & Kersten, 2017).

PPAR‐α is mainly expressed in the liver and controls the expression of genes involved in the uptake and oxidation of fatty acids in this organ (Yan et al., 2023). PPAR‐α mediated responses have been extensively studied in the liver, where activation of this receptor leads to the expression of genes that regulate lipid oxidation (Liang et al., 2022). Numerous studies in animal models with IR have demonstrated that PPAR‐α agonists significantly reduce TG content and adiposity in the liver (Guerre‐Millo et al., 2000). PPAR‐α also regulates the expression of genes encoding several mitochondrial enzymes involved in fatty acid catabolism and mediates the induction of mitochondrial and peroxisomal fatty acid β‐oxidation, establishing its role in maintaining fatty acid balance (Aoyama et al., 1998). Stearoyl‐CoA desaturase‐1 (SCD1) is an important enzyme that regulates lipid metabolism and plays a role in liver lipid metabolism. SCD1 converts saturated fatty acids (SFAs) into mono‐unsaturated fatty acids (MUFAs), which are incorporated into TGs and stored in lipid droplets, protecting liver cells from lipotoxicity. Inhibition of SCD1 expression in liver cells leads to increased AMPK activity and lipophagy, as well as reduced lipid accumulation. SCD1 also regulates lipophagy through AMPK to influence lipid metabolism in primary liver cells from mice. Furthermore, SCD1 is involved in regulating the transcription of the Scd1 gene and maintaining intracellular lipid balance in liver cells exposed to a lipotoxic environment (Sinha et al., 2017; Zhang et al., 2020; Zhou et al., 2020). Mice with a specific deficiency in hepatic SCD‐1 are protected from carbohydrate‐induced hepatic steatosis (Miyazaki et al., 2007). Previous research has suggested that individuals with NAFLD have increased SCD1 activity and that deletion of the SCD1 gene reduces liver lipid synthesis, indicating a close relationship between SCD1 and NAFLD (Kotronen et al., 2009; Ntambi et al., 2002; Puri et al., 2009). Xu et al. showed that administering 500 mg/kg of pomegranate flower extract (PFE) to rats with type 2 diabetes and obesity improved their fatty liver condition. The authors suggest that PFE increases the expression of genes regulated by PPAR‐α and SCD‐1 in the liver. These genes are involved in fatty acid oxidation. The results suggest that clinical trials could provide evidence for the efficacy of PFE in the prevention and treatment of NAFLD caused by diabetes and obesity. This is achieved by regulating abnormal lipid metabolism (Xu et al., 2009). TNF‐α is a pro‐inflammatory cytokine that plays a role in the pathophysiology of NAFLD by inducing IR and inflammation. Previous research has shown that pomegranate juice can reduce TNF‐α levels (Sohrab et al., 2014). TGF‐β is a family of essential cytokines that regulate cell growth and development, inflammation, and processes such as angiogenesis and regeneration (Landström & Liu, 2019). TGF‐β is involved in the development of NAFLD, particularly in the progression of fibrosis (Koeck et al., 2014). TGF‐β is a pro‐fibrotic cytokine that stimulates the production of extracellular matrix proteins and inhibits their degradation, leading to fibrosis (Zhao et al., 2022). In NAFLD, TGF‐β levels are increased and contribute to the progression of the disease from simple steatosis to NASH and fibrosis (Fabregat & Caballero‐Díaz, 2018).

**FIGURE 2:**
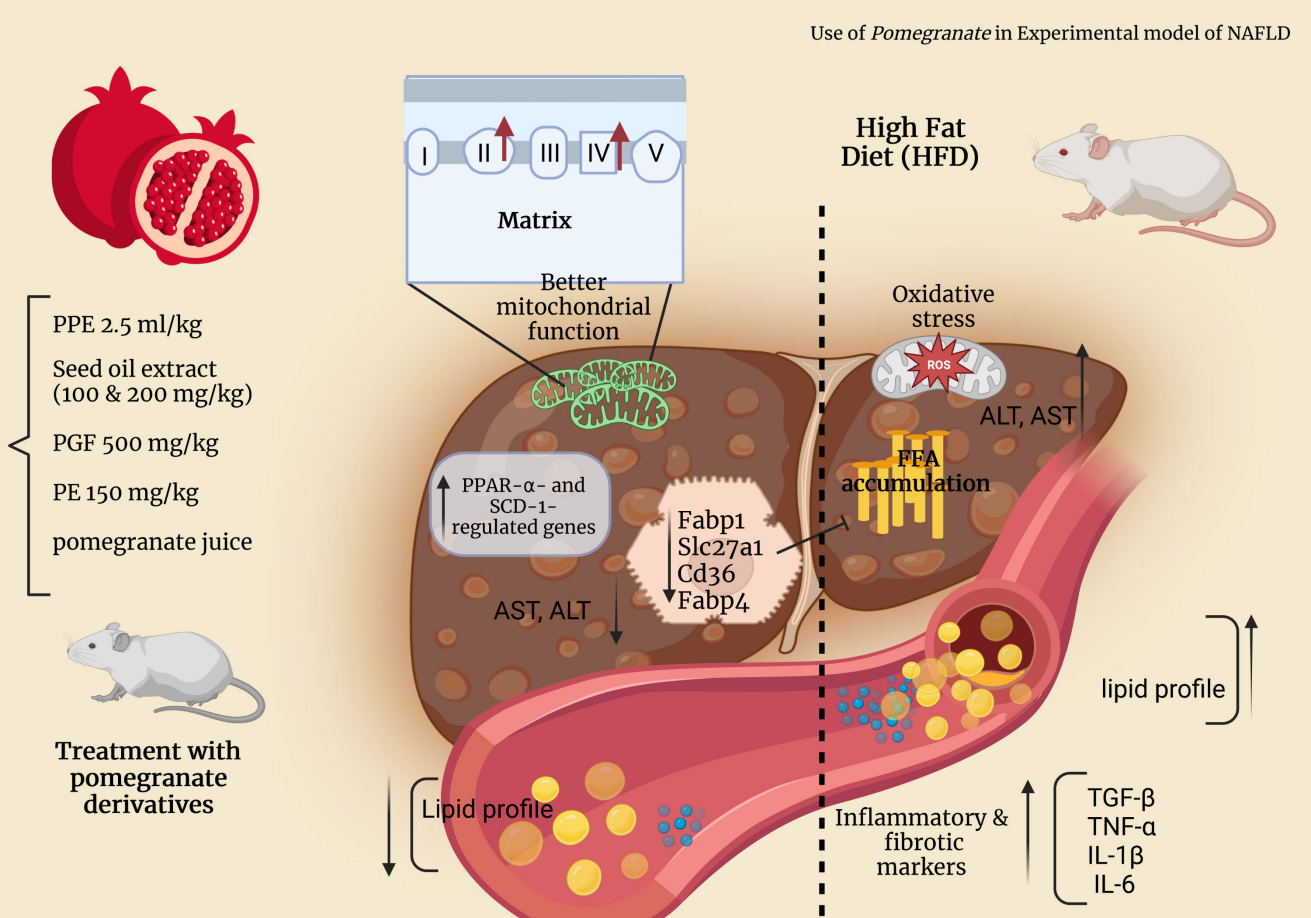
Effects of pomegranate (*Punica granatum*) on NAFLD: Experimental studies.

Noori and colleagues found that pomegranate juice (PJ) reduced hepatic inflammation and fibrosis through several mechanisms. Firstly, PJ consumption decreased the expression of pro‐inflammatory cytokines, such as TNF‐α, IL‐1β, and IL‐6 in the liver. Secondly, PJ increased the expression of anti‐inflammatory cytokines, such as IL‐10 (an anti‐inflammatory mediator). By upregulating IL‐10 expression, PJ further dampens the inflammatory cascade and reduces hepatic inflammation. Thirdly, PJ consumption decreased the expression of TGF‐β in the liver. By reducing TGF‐β expression, PJ helps to inhibit the activation of hepatic stellate cells, which are responsible for the excessive deposition of extracellular matrix and the progression of fibrosis. In summary, pomegranate juice (PJ) reduces hepatic inflammation and fibrosis through several mechanisms. It downregulates pro‐inflammatory cytokines and upregulates anti‐inflammatory cytokines in the liver. PJ also inhibits the expression of TGF‐β, a profibrotic cytokine. Additionally, the polyphenols in PJ have antioxidant properties. Through these combined effects on inflammation, cytokine balance, fibrosis signaling, and oxidative stress, PJ is able to attenuate liver inflammation and fibrosis associated with NAFLD (Noori et al., 2017). We presented a summary of the results of the studies in Figure 2 and Table 1.

**TABLE 1:** Experimental studies consist of the purpose of the present review.

Authors	Dosage	Parts of pomegranate	Type of animal model	Mechanisms
Al‐Shaaibi et al.	15 g dry solids/100 mL	Pomegranate peel extract	Rat	Pomegranate peel extract improved the appearance of their livers, decreased their body weight, improved their liver enzyme levels, and improved the cellular redox status by increasing the levels of GSH in the liver tissue
Poonam and Rao	100 & 200 mg/kg	Pomegranate seed oil extract	Rat	Pomegranate seed oil extract improved antioxidant levels and reduced lipid peroxidation in treated animals
Ayad et al.	20 g of dry powder to 1000 mL of boiled distilled water	Pomegranate peel extract	Guinea pig	Pomegranate peel extract reduced the serum lipid profiles, as well as an improvement in liver function as indicated by the reduction in ALT and AST levels
Zou et al.	150 mg/kg/day	Pomegranate husk extract	Rat	Pomegranate husk extract reduced obesity development and improved serum parameters such as cholesterol, adiponectin, and leptin. Punicalagin reduced oxidative stress in hepatocytes by activating Nrf2
Y‐ Wei et al.	(75, 150, 300 mg/kg)	Pomegranate flower polyphenols	Rat	Compared with model group, pomegranate flower polyphenols significantly decreased fat in liver cells, increased the antioxidant capacity, and significantly increased the expression level of PON1 mRNA and protein in the liver
Xu et al.	500 mg/kg	Pomegranate flower extract	Rat	Pomegranate flower extract improved the fatty liver in rats with type 2 diabetes and obesity. It is possible that the herb‐induced increase in the hepatic expression of PPAR‐α‐ and SCD‐1‐regulated genes responsible for fatty acid oxidation leads to a decrease in liver lipid accumulation
Noori et al.	PJ was freely available to the treatment group (The diets were prepared weekly and stored as vacuum packed (500 g) at −20°C.)	Pomegranate juice	Rat	Pomegranate juice consumption decreased the expression of pro‐inflammatory cytokines, such as TNF‐α, IL‐1β, and IL‐6 in the liver. Additionally, PJ increased the expression of anti‐inflammatory cytokines, such as IL‐10 (an anti‐inflammatory mediator)

## 7 CLINICAL STUDIES ON THE THERAPEUTIC EFFECTS OF POMEGRANATE (*PUNICA GRANATUM*) ON NON‐ALCOHOLIC FATTY LIVER DISEASE

Recent research reported that several factors contribute to the development of NAFLD. These include IR, the buildup of harmful lipids, and the release of pro‐inflammatory cytokines that can damage the liver and activate hepatic stellate cells, leading to fibrosis (Kuchay et al., 2020). Consuming a diet high in carbohydrates and fat can worsen IR, which is a key factor in the development of NAFLD (Meneghel et al., 2022). OS is a risk factor for NAFLD because it can cause lipid peroxidation in the membranes of liver cells. The resulting oxidation products are harmful and can increase the release of pro‐inflammatory cytokines such as TNF‐a, IL‐6, and fibrinogen. This can lead to inflammation and fibrosis in the liver (Madan et al., 2006). Fetuin‐A is another factor that plays a role in the development of NAFLD. This glycoprotein is produced in the liver and released into the bloodstream. It can interfere with insulin receptor auto‐phosphorylation, leading to IR (Denecke et al., 2003). Additionally, hepatokine can increase the expression of inflammatory cytokines and decrease the adiponectin levels (Hennige et al., 2008). Fibroblast growth factor 21 (FGF‐21) is a hepatokine that can have positive effects on insulin sensitivity and cholesterol levels. However, elevated levels of FGF‐21 have been linked to an increased risk of cardiovascular disease and IR. High levels of FGF‐21 in the blood can be an indicator of NAFLD, even when other risk factors are not present. Furthermore, the level of FGF‐21 in the blood is strongly and independently associated with liver fat content, markers of liver cell death, and the severity of NAFLD in obese individuals (Francque et al., 2016; Qin et al., 2015). A study by Jafarirad et al. found that taking 450 mg/day of pomegranate extract (standardized to contain 40% ellagic acid) for 12 weeks can reduced liver enzyme levels (ALT and AST), hepatokine levels (fetuin‐A and FGF‐21), and IL‐6 levels while increasing total antioxidant capacity in 44 patients with NAFLD (Jafarirad et al., 2023). Pomegranate is a rich source of antioxidants. However, another study found that HepG2 liver cells treated with pomegranate fruit extract or pure punicalagin were more susceptible to damage from OS than untreated cells (Jafarirad et al., 2023).

HepG2 cells supplemented with pomegranate fruit extract or purified punicalagin were more susceptible to the damaging effects of OS than unsupplemented cells, according to one study (Danesi et al., 2014). Drinking pomegranate juice may have several benefits for people trying to lose weight and protect their livers. One study found that taking pomegranate extract was associated with improvements in cholesterol levels, TG levels, the ratio of LDL‐C to HDL‐C, fasting blood sugar levels, IR, diastolic blood pressure, weight, body mass index (BMI), and waist circumference in patients with NAFLD (Goodarzi et al., 2021). Another study found that drinking pomegranate juice enhanced the effects of aerobic exercise on IR and liver enzyme levels in men with type 2 diabetes (Nemati et al., 2022). In a study, 65 NAFLD patients (average age 39 ± 8 years) drank either 250 mL of pomegranate or orange juice daily for 12 weeks as part of a low‐calorie diet. Both groups saw significant decreases in liver enzyme levels (AST and ALT) and BMI. The pomegranate group also had a significant increase in total antioxidant capacity. This suggests that antioxidant‐rich fruits may benefit NAFLD patients on a low‐calorie diet (Ekhlasi et al., 2015). In a quasi‐study, 35 NAFLD patients were given a weight loss diet and drank 250 mL of pomegranate juice daily for 3 months. Of the 35 individuals who took part in the study, 33 (9 females and 24 males) successfully finished the research. The combination of pomegranate juice and a weight loss diet led to notable reductions in liver enzyme levels and an increase in HDL‐cholesterol (Ekhlasi et al., 2013).

Metabolic syndrome is a cluster of conditions that include abdominal obesity, high blood pressure, high blood sugar levels, and abnormal lipid levels. It is characterized by IR and is a risk factor for cardiovascular disease and type 2 diabetes. The relationship between NAFLD and metabolic syndrome is bidirectional. NAFLD is considered a hepatic manifestation of metabolic syndrome, and the presence of NAFLD increases the risk of developing metabolic syndrome and its associated complications. On the other hand, metabolic syndrome contributes to the development and progression of NAFLD through various mechanisms, including IR, dyslipidemia, and inflammation.

Barghchi et al. conducted a randomized, double‐blind clinical trial with 78 participants to assess the effects of PPE supplementation in people with NAFLD and metabolic syndrome risk factors. The participants received either 150 mg/kg of PPE or a placebo along with a 500 calorie restricted diet for 8 weeks. The results demonstrated that those who received PPE had significant decreases in body weight, waist circumference, BMI, body fat percentage, and trunk fat percentage compared to the placebo group after the 8‐week intervention. Additionally, the PPE group exhibited improvements in lipid profiles, with significant reductions in total cholesterol, TGs, and low‐density lipoprotein cholesterol, and a significant increase in high‐density lipoprotein cholesterol levels. The PPE supplementation was associated with a decrease in fasting blood glucose levels, indicating an anti‐glycemic effect. However, there was no significant change in fasting serum insulin levels. Overall, the study demonstrated that PPE supplementation had beneficial effects on body composition, lipid profiles, blood pressure, glycemic control, and liver health in individuals with NAFLD and metabolic syndrome risk factors (Barghchi, Milkarizi, Belyani, et al., 2023).

Supplementation with pomegranate peel may help alleviate symptoms of depression, anxiety, and stress in patients with NAFLD. This beneficial effect is likely due to the abundance of bioactive compounds in pomegranate peel, including polyphenols, anthocyanidins, tannic acid, gallic acid, and ellagic acid. These compounds have been shown to possess antioxidant properties, which can help protect against OS, a factor associated with depression and anxiety (Kazemabad et al., 2022). Additionally, animal studies have shown that pomegranate peel extract improves depressive behaviors brought on by ongoing stress (Naveen et al., 2013). In a randomized clinical trial, 76 NAFLD patients were divided into pomegranate peel supplementation (*n* = 39) or placebo (*n* = 37) groups. In a study, pomegranate peel (PP) dry extract capsules were prepared using a soaking method. Patients with NAFLD received either 1500 mg of PP extract or a placebo daily for 8 weeks. The results showed that patients taking PP had significantly improved depression and stress scores compared to the placebo group, even after adjusting for potential confounding factors. In summary, supplementing NAFLD patients with 1500 mg per day of pomegranate peel extract for 8 weeks led to reductions in symptoms of depression and stress. This study provides evidence that pomegranate peel supplementation can have beneficial effects on mental health, specifically depression and stress, in patients with NAFLD (Barghchi, Milkarizi, Dehnavi, et al., 2023).

**FIGURE 3:**
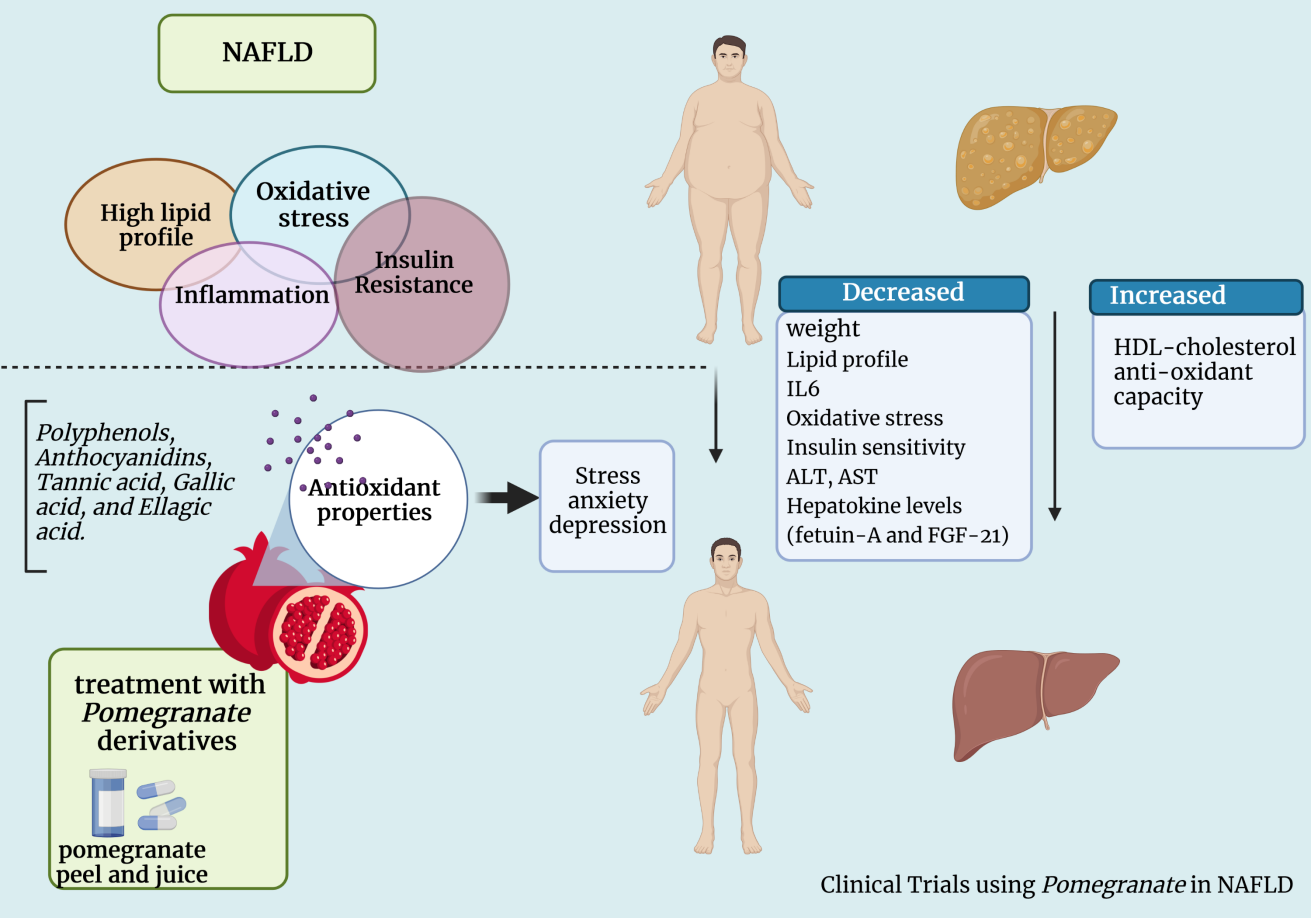
Effects of pomegranate (*Punica granatum*) on NAFLD: Clinical studies.

Overall, regular consumption of pomegranate or its extracts may have positive effects on NAFLD by reducing liver fat, improving liver function, reducing inflammation, enhancing insulin sensitivity, and preventing OS. However, it is important to note that individual results may vary, and pomegranate should not be considered a standalone treatment for NAFLD. Consulting a healthcare professional is advisable for personalized advice and management. We presented a summary of the results of the studies in Figure 3 and Table 2.

**TABLE 2:** Clinical studies consist of the purpose of the present review.

Authors	Dosage	Form of pomegranate	Type of study	Mechanisms
Jafarirad et al.	450 mg/day	Pomegranate extract tablets	Randomized double blind clinical trial	Pomegranate extract tablet reduced liver enzyme levels (ALT and AST), hepatokine levels (fetuin‐A and FGF‐21), and IL‐6 levels while increasing total antioxidant capacity
Ekhlasi et al.	250 mL/day	Pomegranate juice	Randomized clinical trial	Pomegranate juice decreased liver enzyme levels (AST and ALT) and BMI, and also increased total antioxidant capacity
Ekhlasi et al.	250 mL	Pomegranate juice	Quasi‐experimental trial	Pomegranate juice decreased liver enzyme levels and an increased in HDL‐cholesterol
Barghchi et al.	150 mg/kg/day	Pomegranate peel extract	Randomized double‐blind clinical trial	Pomegranate peel extract supplementation had beneficial effects on body composition, lipid profiles, blood pressure, glycemic control, and liver health in individuals with NAFLD and metabolic syndrome risk factors
Barghchi et al.	1500 mg	Pomegranate peel	Randomized clinical trial	8‐week pomegranate peel supplementation has ameliorating effects on depression and stress symptoms in NAFLD patients

## 8 CONCLUSION

NAFLD is a common chronic liver disease characterized by the accumulation of fat in the liver in the absence of excessive alcohol consumption. The pathogenesis of NAFLD involves multiple factors, including IR, inflammation, OS, and hepatokine dysregulation. In the development of NAFLD, both the OS and inflammation play crucial roles. OS leads to lipid peroxidation in liver cell membranes, causing hepatocyte injury and inflammation. Inflammation further promotes the progression of NAFLD, leading to fibrosis and liver damage. Recent studies have suggested that dietary components or medicinal plants with pharmacological capabilities could be used as alternative treatments for NAFLD.

The researchers chose pomegranate (various parts such as fruit, seed, peel, and flower) as a potential treatment for NAFLD due to its rich content of various phytochemicals such as polyphenols. For example, PPE possesses potent antioxidant properties due to its high content of phenolic and flavonoid compounds. By reducing OS, PPE helps to prevent excessive cellular damage and improves liver function. Additionally, PPE can help to regulate lipid metabolism and prevent the accumulation of fat in hepatocytes, which is a characteristic feature of NAFLD. Pomegranate juice is also rich in antioxidants, which can aid in the prevention of OS and inflammation associated with NAFLD. Additionally, pomegranate fruit extract tablet can reduce lipid peroxidation, inhibit pro‐inflammatory cytokines, and improve mitochondrial function, all of which contribute to an increase in TAC.

Overall, the results of this study suggest that pomegranate extract (fruit, seed, peel, etc.) has potential therapeutic benefits for NAFLD patients. However, further research is needed to fully understand its mechanisms of action, establish optimal dosage and treatment duration, and assess long‐term outcomes.

## AUTHOR CONTRIBUTIONS

**Mohammad Yasin Zamanian:** Conceptualization (equal); project administration (lead); visualization (equal); writing – original draft (equal); writing – review and editing (equal). **Mehraveh Sadeghi Ivraghi:** Conceptualization (equal); writing – original draft (equal). **Lusine Khachatryan:** Data curation (equal); resources (equal). **Diana Vadiyan:** Data curation (equal); resources (equal). **Hanie Bali:** Methodology (equal); validation (equal); writing – original draft (equal). **Maryam Golmohammadi:** Conceptualization (equal); validation (equal); visualization (equal); writing – original draft (equal); writing – review and editing (equal).

## CONFLICT OF INTEREST STATEMENT

The authors declare that they do not have any conflict of interest.

## Data Availability

The data presented in this study are available in the article.
